# CCL21 Cancer Immunotherapy

**DOI:** 10.3390/cancers6021098

**Published:** 2014-05-07

**Authors:** Yuan Lin, Sherven Sharma, Maie St. John

**Affiliations:** 1 Department of Head and Neck Surgery, David Geffen School of Medicine at UCLA, 10833 Le Conte Avenue, Los Angeles, CA 90095, USA; 2 Jonsson Comprehensive Cancer Center, David Geffen School of Medicine at UCLA, 10833 Le Conte Avenue, Los Angeles, CA 90095, USA; 3 UCLA Head and Neck Cancer Program, David Geffen School of Medicine at UCLA, 10833 Le Conte Avenue, Los Angeles, CA 90095, USA; 4 Clinical and Translational Science Institute, David Geffen School of Medicine at UCLA, 10833 Le Conte Avenue, Los Angeles, CA 90095, USA; 5 Division of Pulmonary and Critical Care Medicine, Department of Medicine, David Geffen School of Medicine at UCLA, 37-131 CHS, 10833 Le Conte Avenue, Los Angeles, CA 90095, USA; 6 Veterans’ Affairs Greater Los Angeles Healthcare System, Los Angeles, CA 90073, USA

**Keywords:** CCL21, cancer immunotherapy, dendritic cell, polymer, T cell

## Abstract

Cancer, a major health problem, affects 12 million people worldwide every year. With surgery and chemo-radiation the long term survival rate for the majority of cancer patients is dismal. Thus novel treatments are urgently needed. Immunotherapy, the harnessing of the immune system to destroy cancer cells is an attractive option with potential for long term anti-tumor benefit. Cytokines are biological response modifiers that stimulate anti-tumor immune responses. In this review, we discuss the anti-tumor efficacy of the chemotactic cytokine CCL21 and its pre-clinical and clinical application in cancer.

## 1. Introduction

Chemokines are cellular cytokines involved in leukocyte migration, activation and regulation of angiogenesis. They also play a functional role in the maintenance of immune homeostasis and the architecture of secondary lymphoid organ. Secondary lymphoid chemokine (CCL21) (thymus-derived chemokine 4, 6Ckine or Exodus 2) is produced by several cell types that include high endothelial venules, lymphoid endothelial cells as well as the stromal cells within T cell areas of lymph nodes, spleen, and Peyer’s patches. CCL21 mediates its activity through the G-protein coupled CCR7 transmembrane receptor. In the T cell zones of secondary lymphoid organs, T-cell activation is facilitated by CCL21 following the recruitment and co-localization of naive lymphocytes and antigen stimulated dendritic cells (DC) [1,2]. CCL21 molecules are bound to the surface of lymph node DC. Contact with antigen-presenting cells (APC) bearing CCL21 chemokine co-stimulates T cells by a two-step contact mechanism. T cells initially form an antigen-independent “tethered” adhesion on CCL21-bearing antigen-presenting cells. The formation of these tethers supersedes T cell receptor signaling and immunological synapse formation. However, chemokine-tethered T cells are hyper-responsive to subsequent contacts with antigen-presenting cells. Thus, T cells are co-stimulated “in trans” and sequentially after initial engagement with their CCL21-rich environment [3]. In addition to inducing chemotactic migration, CCL21 co-stimulates expansion of CD4^+^ and CD8^+^ T cells and induces Th1 polarization. The immune suppressor cell population, CD4^+^CD25^+^ regulatory T (Treg) cells are hyporesponsive to CCL21 induced migration, and unresponsive to CCL21 co-stimulation. These functions of CCL21 to both attract naïve T cells as well as co-stimulate their proliferation, differentiation and activation suggests that CCL21 plays a significant role in initiating T cell responsiveness that increases the therapeutic relevance for paracrine delivery of CCL21 [4]. The anti-tumor effectors NK and NKT cell subsets also express the CCR7 receptor and are chemo attracted by CCL21.

## 2. CCL21 and Anti-Tumor Immunity

CCL21 priority ranking is 13 amongst the list of twenty National Cancer Institute ranked biological agents with high potential for use in cancer therapy. The role of CCL21/CCR7 axis in tumor immunity has been closely investigated in the past two decades. The ability of CCL21 to chemoattract DC and T lymphocytes forms the rationale for CCL21-based cancer immunotherapy. One of the challenges in developing immunotherapy for cancer is enlisting the host response to recognize tumors of poor immunogenicity. Effective anti-tumor responses require APC, lymphocyte and NK effectors. Although cancer cells express tumor antigens, the limited expression of major histocompatibility complex (MHC) antigens, defective transporter associated with antigen processing, and lack of co-stimulatory molecules make them ineffective APC. Effective anti-cancer immunity can be achieved by recruiting professional host APC for tumor antigen presentation to promote specific T-cell activation. DCs serve as natural APC and initiate cellular T cell immune responses [5]. DCs acquire tumor antigens and migrate to T cell areas of lymphoid organs for priming specific anti-tumor T cell activity. CCL21 chemokine attracts and co-localizes naive lymphocytes and antigen stimulated DC into T cell areas of lymphoid organs, that eventuate in cognate T cell activation [1]. Thus, the ability of CCL21 to promote T cell activation through the cognate interaction of recruited T lymphocytes and DC has potential to circumvent tumor-mediated immune suppression and orchestrate effective T cell-mediated anti-tumor activity.

The Dubinett and Sharma group first proposed and demonstrated that the establishment of CCL21 chemotactic gradient that favors localization of activated DC within the tumor is an effective strategy to restore antigen presentation [6]. They demonstrated that intratumoral injection of recombinant CCL21 mediated T cell dependent anti-tumor response in a syngeneic immune competent model of lung cancer. In immune competent mice, injection of CCL21 induced infiltration of CD4 and CD8 T cell and DC in both tumor and draining lymph nodes [6]. The requirement of both CD4 and CD8 T cell subsets for CCL21 mediated tumor inhibition was confirmed in CD4 and CD8 T cell knockout mice [7]. CCL21 mediated immune response included an increased influx of CD4 and CD8 T cells and DC in the tumor, as well as increased IFN-γ, MIG-CXCL9, CXCL10, GM-CSCF and IL-12, but a concomitant decrease in immunosuppressive molecules such as PGE-2 and TGF-β [8]. Further studies showed that the full potency of CCL21-mediated anti-tumor response required the induction of IFN-γ, MIG/CXCL9 and IP-10/CXCL10 in concert, as neutralization or depletion of any one of these cytokines led to a decrease in the frequency of CXCR3+ve T cells and CD11c+ve DC in the tumor [8,9]. Injection of CCL21 also generated systemic immune response, because splenic lymphocytes from CCL21 treated tumor-bearing mice demonstrated enhanced cytolysis against autologous tumors [6]. The anti-tumor efficacy of CCL21 was further confirmed in a transgenic murine model of lung cancer [10]. Recently intratumoral injection of vault nanocapsule packaged CCL21 increased infiltration of DC and T lymphocyte effectors and reduced myeloid derived suppressor cells (MDSC) and T regulatory cells [11]. Studies by other investigators confirmed the anti-tumor activity of CCL21 in several tumor models. For example, CCL21 and other lymphocyte specific chemokines such as EBI-1-ligand chemokine (ELC) and stromal cell-derived factor (SDF)-1alpha, showed anti-tumor immunity both systemically and locally in fibrosarcoma and ovarian tumor via activation of type 1 T cell response and IFN-γ generation [12]. CCL19 also promoted a T cell dependent anti-cancer response in a murine lung cancer model [13]. In this study, transfer of T lymphocytes from CCL19 treated tumor-bearing mice conferred the anti-tumor therapeutic efficacy of CCL19 to naive mice [13]. In summary, these studies demonstrated that CCL21 has the capacity to recruit anti-tumor lymphocytes and APCs in tumor tissue, resulting in tumor regression or rejection. 

In studies by Shields *et al.* in the B16-F10 melanoma model, tumor secreting CCL21 led to induction of lymphoid neogenesis and recruitment of regulatory T cells and MDSC cells [14]. Melanoma expressing high CCL21 level by lentivirus (~0.18 pg/1000 cells *in vitro*) displayed more aggressive growth at day 9 and exhibited immunosuppressive microenvironment, which were absent in melanoma with low levels of CCL21 caused by shRNA knockdown [14]. CCL21 secreting B16-F10 melanoma cells and related tumor environment seemed to recruit functional activated DCs, while promoted differentiation of naïve T cells to Treg cells, which was normally induced by immature DC [14]. In contrast, in another C57BL/6 mouse model of melanoma based on B16F0 melanoma cell line, high level CCL21 release (~800 pg/5 × 10^5^ cells/48 h *in vitro*) driven by UB promoter from melanoma enabled massive infiltration of tumors with CD4^+^CD25^−^, CD8^+^ T lymphocytes, and CD11c^+^ dendritic cells, and consequent activation of cellular and humoral immune responses sufficient for complete rejection of CCL21-positive melanomas within 3 weeks in all tumor-inoculated mice [15]. Low level CCL21 release (~120 pg/5 × 10^5^ cells/48 h *in vitro*) driven by CMV promoter from melanoma led to smaller, but not rejected, tumor growth after 3 weeks. In Novak’s model, there was no significant difference in tumor growth between all groups before day 11 [15]. The different growth course of tumor in above two experiments may be explained by the different cancer cells used and the different CCL21 amount released in the study. B16F0 and B16F10 may not relate to each other, according to ATCC [16]. B16F10 is lung-specific highly-metastatic clone selected following *in vivo* passaging [17], whereas B16F0 is a low-malignant weakly-metastatic melanoma clone [18]. Thus, B16F0 cells grow slower than B16F10 cells *in vivo*. The intrinsic difference in malignancy and metastasis of these two cells may also regulate host-tumor immune interactions. It is plausible that high-malignant cancer cells could inhibit DC maturation and cytokine stimulation. In addition, in hepatocellular carcinoma model, Liang *et al.* showed that tumor secreting CCL21 led to a significant delay of tumor progression, as well as a profound tumor infiltration of DCs and activated CD4(+) T cells and CD8(+) T cells [19]. Local expression of CCL21 by cancer cells also reduced tumor growth in nude mice, probably through inhibition of neoangiogenesis [19]. In animal model of prostate cancer, local expression of CCL21 by cancer cells controlled by tet-on system enhanced lymphocytes infiltration, inhibited tumor growth and metastasis [20]. Shields *et al*. noted that the CCL21 produced endogenously by the B16-F10 melanoma induced lymphoid like stroma and immune escape by tumors. However, in a recent study identification of ectopic lymph node or tertiary lymphoid structures within human non-small cell lung cancer correlated with better long term survival in patients [21]. Nonetheless, we believe caution should be exercised in interpreting the implications of CCL21 in tumor growth and anti-tumor immunity, especially given the ongoing clinical trials of CCL21 in lung [22] and melanoma patients. The results of the studies of CCL21 on anti-tumor immunity suggest that interaction between tumor released CCL21 and host immune response is complex. The induction of immune response may be tumor/environment-specific or dependent on timing and amount of CCL21 released. Taken together, majority of discoveries support a role of CCL21 in anti-tumor immunity, however concerns remain that tumor-derived CCL21 may induce immune tolerance to tumor antigens in some models.

In accord with basic research, recent clinical observations also supported the anti-tumor role of CCL21. A clinical study showed that high tumor infiltration by cytotoxic CD8^+^ T cells expressing CCR7 had a favorable prognostic value in colon cancer patients [23]. In contrast, patients whose tumor tissue presents a combined low infiltration score for both of CCR7+ and regulatory lymphocyte cell populations had a very poor outcome [23]. A similar finding was observed in renal cell carcinoma patients. Local expression of CCL21 by tumor tissue induced accumulation of mature DCs and proliferating T-cells at the tumor margin, reflecting a local anti-tumor immune response [24]. This suggests that the recruitment of DCs in patient tumor tissue could be achieved by local expression of CCL21.

## 3. Genetic Modified Dendritic Cell in CCL21 Immunotherapy

Dendritic cells are potent antigen presenting cells to stimulate naïve T cells in immune system [25]. DCs are bone marrow derived cells and located throughout the body as sentinels. Following encounter with foreign or tumor antigen, DCs become activated (mature), capture antigen, migrate to lymphoid tissues in response to chemokines such as CCL21, and undergo physiological change to present antigen in association with major histocompatibility complex (MHC) to T cells. In contrast, non-activated (immature) DCs present antigens to T cells that lead to Treg cells differentiation and immune tolerance [26]. Thus, DCs play a central role in the modulation of immune activity [27]. The crucial role of DCs in immune activation makes DC an important candidate for adoptive cell transfer in cancer immunotherapy [28].

For practical purposes, protocols for *ex vivo* DC culture and maturation from peripheral blood and hematopoietic precursors have been developed and improved [29,30], thus allowing for tumor antigen pulsing or genetic modification of DCs for enhanced anti-tumor function. For example, human peripheral blood mononuclear leukocytes differentiated from monocytes in GM-CSF and IL-4 generate enriched and mature DCs [31]. The mature patient-derived DCs can be educated to recognize specific tumor associated antigen (TAA) and infused back to patients to launch anti-tumor immune response. In 2010, first DC based cancer immunotherapy Sipuleucel-T invented by Dendreon was approved by FDA for the treatment of metastatic hormone-refractory prostate cancer [32]. Based on clinical trials, DC-based cancer vaccines have proven to be safe and non-toxic [33].

Although DCs have been translated to the clinic in immunotherapeutic trials, one of the hurdles for DC based immunotherapy is that the maturation state of DC is not always satisfactory [34]. Original protocol for DC maturation [35] does not stimulate efficient Th1 cells *in vivo* as the DCs are deficient in pro-anti-tumor cytokine production, but rather induce Treg cells [34]. Several approaches to generate DC with enhanced immune stimulatory capabilities include the transduction of genes/mRNAs to increasing cytokine and chemokine production for DC trafficking and T cell priming. T-cell activity can be increased by activating the DCs through electroporation with mRNA encoding CD40 ligand, CD70, and a constitutively active Toll-like receptor 4 (TriMix DCs) [36,37,38]. In addition, DCs that ectopically express cytokines and chemokines live longer and/or are more immune stimulatory. These DCs can be pulsed with tumor antigens *ex vivo* or *in vivo* to target cancer cells. Several cytokines have been studied for this purpose. IL-12 is responsible for Th1 polarization of lymphocytes. DC over expressing IL-12 effectively present antigens to T cells that led to tumor regression [39,40,41,42,43]. DC transduced with other type 1 cytokines such as IL-2 or IL-7 also exhibited similar anti-tumor effect [39,44,45]. Genetically modified DCs expressing GM-CSF and pulsed with antigens have demonstrated a strong anti-tumor response [46].

Besides cytokines, DCs can be activated by viral vector transduction of chemokine CCL21, leading to enhanced antigen presenting ability and lymphocyte trafficking [47]. In most of these studies, adenovirus or adeno associated virus were used. Adenovirus exhibits high level transgene expression and does not require cell division for infection. Adeno associated virus exhibits low host immunity [48]. In one study, DCs transduced with adenoviral CCL21 (AdCCL21) at multiplicities of infection (MOIs) of 50:1 or 100:1 were able to produce up to 210 ± 9 ng/mL and 278 ± 6.5 ng/mL human CCL21 per 10^6^ cells per 48 h *in vitro*, respectively [49]. After transduction, DCs kept immature phenotype in culture, while the supernatant chemo attracted mature DC and activated lymphocytes [49]. The intratumoral injection of the DC-CCL21 would potentially capture tumor antigens, present it to recruited lymphocytes and launch an enhanced immune response to destroy cancer. In this method, an *in situ* source of tumor antigen is presented to DCs, compared to *in vitro* immunization by purified tumor antigen. In addition, compared to direct intratumoral administration of recombinant CCL21, DC-CCL21 approach obviates high and frequent dosing and limits CCL21 diffusion and degradation of bolus administration. In addition, DC-CCL21 approach removes the unnecessary systemic toxicity and cost of recombinant CCL21 administration. Furthermore, DC-CCL21 approach could be used as adjuvant treatment for standard cancer therapy.

In murine model of melanoma, intratumoral injections of DC-CCL21 three times in two weeks led to tumor growth inhibition that was significantly better than either control DCs or CCL21 alone. Distal site immunization of tumor-bearing mice with DC-CCL21 pulsed with tumor lysate elicited an anti-tumor response with migration of T cells to the immunization site [47]. The anti-tumor efficacy of DC-CCL21 was later explored in murine lung cancer models. 60% of mice treated with DC-CCL21 weekly for three weeks showed complete lung tumor eradication, in contrast, only12% mice treated with controlled DC showed tumor rejection and mice with recombinant CCL21 injection at similar dose showed no anti-tumor effect [9]. DC-CCL21 administration led to increases in the CD4^+^, CD8^+^, CD3^+^CXCR3^+^ T cells and DC expressing CD11c^+^DEC205^+^, as well as induction of Th1 cytokines, whereas CD4^+^CD25^+^ T-regulatory cells were markedly reduced. DC-CCL21 mediated anti-tumor responses required IFN-γ, MIG/CXCL9, and IP-10/CXCL10 [9]. These studies demonstrate that autologous tumor is able to provide injected DCs access to the entire repertoire of antigen *in situ*, thus increasing the success rate and reducing potential immune tolerance due to phenotypic modulation [9]. Based on these observations, anti-tumor efficacy of DC-CCL21 was evaluated in a clinical relevant transgenic mouse model of lung adeno carcinoma with an average life span of 4 months. DC-CCL21 treatment led to reduced tumor burden compared to control DC and recombinant CCL21 groups. Median survival was 24 ± 1 weeks for DC-CCL21 treated mice, compared to 18 ± 2 weeks for control groups [50].

The success of DC-CCL21 therapy in animal cancer models provided a strong rationale for evaluation of DC-CCL21 in cancer immunotherapy. The density of mature DCs is a good predictor of NSCLC patient survival and clinical outcome [21]. Circulating mature DCs are decreased in lung cancer patients, thus intratumoral administration of DC could be a promising approach to alleviate this problem. Based on the promising results of extensive pre-clinical testing of intratumoral DC-CCL21 in murine tumor models, a phase 1 clinical trial of DC-CCL21 was initiated in late stage non-small cell lung cancer patients at University of California Los Angeles. The safety and clinical activities of the intratumoral administration of autologous DC-CCL21 were evaluated in patients with pathologically confirmed and radiographically measurable NSCLC (Stage IIIB/IV) who had tumor accessible by CT-guided or bronchoscopic intervention, and were refractory to standard therapy [7]. Findings from this trial thus far suggest that intratumoral administered DC-CCL21 vaccine is safe with no associated adverse reactions at the doses administrated (1 × 10^6^, 5 × 10^6^, 1 × 10^7^ , or 3 × 10^7^ DC-CCL21 cells/injection) and with systemic anti-tumor specific immune responses elicited [51]. In Moffitt Cancer Center, a phase I clinical trial was initiated to assess the toxicity, immune responses, and anti-tumor clinical responses in human leukocyte antigen-A*0201-positive patients with chemotherapy-naive metastatic melanoma receiving escalating doses of adenoviral CCL21-transduced DC matured *ex vivo* with a cytokine cocktail and pulsed with MART-1/gp100/NY-ESO-1 class I peptides and keyhole limpet hemocyanin [52]. Early data from these studies show the known chemotactic selectivity of CCL21 with the accumulation of T cells in biopsies from one of the melanoma injection sites.

## 4. Polymer-Delivered CCL21 Immunotherapy

The science of biomaterial engineering for drug delivery has evolved considerably for the past 30 years. Novel technology allow to design functional, biocompatible and biodegradable polymer vehicles, such as poly-ε-caprolactone (PCL), poly (lactide-co-glycolide) (PLG), as well as alginate and fibrin hydrogel, for molecular and cellular delivery in cancer immunotherapy [53]. Three dimensional porous polymer scaffolds exhibit great ability to deliver cytokine molecules and immune cells with spatiotemporal specificity, to promote cell-cell interaction in matrix and, to direct cell function [53]. This ability forms the rationale for polymer-based CCL21 cancer immunotherapy that could program host immune cells *in vivo*. These materials can be further integrated with other anti-cancer treatments in the design of next-generation therapy against cancer [54].

PCL/PLCL co-polymer loaded with DC-CCL21 or chemotherapy drug cisplatin has been tested in an animal model of Head and Neck Squamous Cell Carcinoma (HNSCC) to prevent cancer recurrence [55,56]. HNSCC is difficult to resect completely by surgery due to complicated context and therefore exhibits high recurrence rate in the patients [57]. A drug delivery platform with spatiotemporal specificity is in demand for anti-recurrence therapy. In order to accomplish these requirements, a polymer platform was made from a mixture of a ratio of 70:30 of PCL to PLCL with relevant amount of CCL21 and/or cisplatin and was spread on a glass to form a thin sheet. The final product is a flexible sheet that exhibits nice drug release kinetics and can adhere to the surgical resected tissue contours [55]. In the initial animal study, cisplatin loaded PCL/PLCL polymer was applied intraoperatively to the surgical bed after partial tumor resection, replicating the difficult situation seen in patients. The cisplatin secreting polymer effectively reduced tumors by over 16-fold as compared to control plain polymer and intratumoral cisplatin injection groups. When combined with radiation, polymer therapy led to a statistically significant lower tumor weight compared to the radiation alone group and the control group [55]. Based on above data, the PCL/PLCL scaffold was later tested for anti-tumor efficacy of DC-CCL21 therapy. In order to improve DC culture condition for immunotherapy, a thin layer of fibrin hydrogel with 10^6^ DCs seated inside was added to the surface of PCL/PLCL polymer [56]. The component of hydrogel and polymer was optimized for the maximum production of bioactive CCL21. After implantation to the partially resected tumor, the gradient of local CCL21 that resulted from its sustained and localized release led to the recruitment of CD4+ T cells and CD11c+ DCs into the tumor, while tumor infiltrating Treg cells were decreased. Overall, DC-CCL21 polymer treatment significantly reduced tumor burden, compared to control DC group or recombinant CCL21 injection group [56]. Currently, anti-tumor efficacy of polymer loaded with recombinant CCL21 is being further evaluated with combination of cisplatin chemotherapy and radiation therapy.

In addition to cytokines and immune cells, tumor associated antigen can also be loaded in polymer to activate DCs. Subcutaneous implantation of PLG polymer loaded with cytokine GM-SCF, TLR agonist CpG and tumor lysate as antigen led to host DC recruitment, activation and subsequent homing to lymph nodes [58]. This vaccine induced 90% prophylactic tumor protection and therapeutic protection. The polymer scaffold also displayed long term activity for months post implantation, which is superior to all soluble administration methods to date [58].

Other biomaterials, such as vault nanoparticles, were also investigated for intratumoral CCL21 delivery. In a well characterized Lewis lung cancer model, CCL21-vault nanoparticles system showed effective anti-tumor efficacy. A single intratumoral injection of CCL21-vault nanoparticles was able to recruit anti-tumor effectors that induced potent anti-tumor activity and inhibit tumor growth [11]. The nanoparticle system can be further designed for target delivery and specific payloads to prime the immune system.

## 5. Conclusions

**Figure 1:**
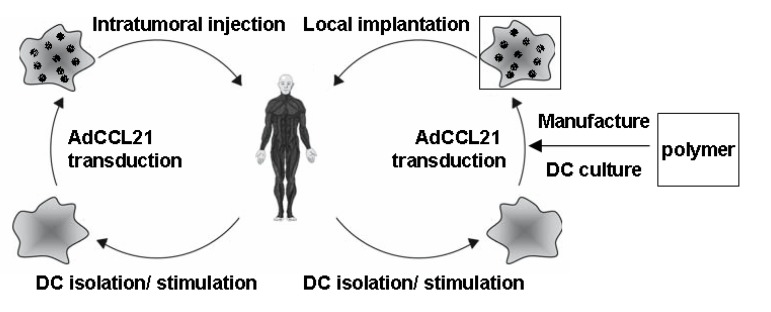
DC-CCL21 Immunotherapy (**Left**) Patient-DCs are isolated from PBMC and stimulated with 800 U/mL GM-CSF and 400 U/mL IL-4 for 6 days. Transduction with clinical grade adenoviral vector encoding CCL21 generates CCL21 secreting DCs (DC-CCL21), which are injected into patient’s tumor; (**Right**) DC-CCL21 cultured in fibrin hydrogel in PCL-PLCL polymer platform overnight for potential implantation in the tumor following surgical resection (proposed clinical trial for HNSCC).

**Figure 2:**
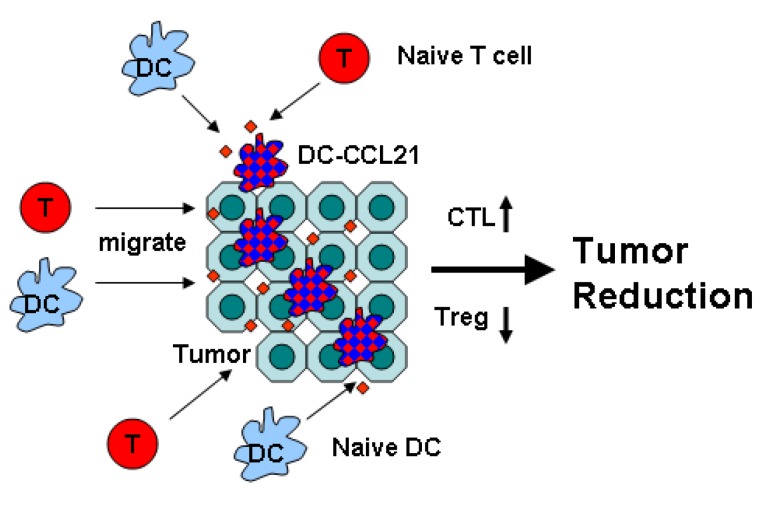
Mechanisms of DC-CCL21 Anti-Tumor Activity Intratumoral administration of DC-CCL21 promotes chemo-taxis of naïve T cell and DC into the tumor and increases APC activity of DC leading to activation of cytotoxic T lymphocytes (CTL) but reducing activity of regulatory T cell (Treg) that culminate in tumor reduction.

The past 20 years of accumulated research evidence clearly indicates a fundamental role of CCL21 in anti-tumor immune activity. CCL21 is critical in mediating activated DCs and T cells to lymph system niches and triggering subsequent immune response to foreign antigen, therefore providing the rationale for CCL21 based cancer immunotherapy. The results of preclinical animal cancer models and phase 1 DC-CCL21 trial in lung cancer and melanoma patients are promising. Preclinical findings suggest that improved anti-tumor efficacy of CCL21 treatment can be achieved by combining with radiation, chemotherapy or targeted therapy regimens. Figure 1 and Figure 2 show the strategy of intratumoral CCL21 delivery by DC against cancer and the ensuing activities on naïve T cell and DC recruitment into the tumor leading to T cell activation and tumor reduction. DC-CCL21 immune efficacy may further be enhanced by T cell exhaustive marker blockade to maintain long term anti-tumor CTL function in the tumor microenvironment. Material engineering provides several attractive strategies to design more potent CCL21 immunotherapy. Collaboration among immunologists, material scientists and physicians will create future generations of CCL21 based anti-cancer immunotherapy.
